# Unsupervised Learning for Monaural Source Separation Using Maximization–Minimization Algorithm with Time–Frequency Deconvolution †

**DOI:** 10.3390/s18051371

**Published:** 2018-04-27

**Authors:** Wai Lok Woo, Bin Gao, Ahmed Bouridane, Bingo Wing-Kuen Ling, Cheng Siong Chin

**Affiliations:** 1 School of Electrical and Electronic Engineering, Newcastle University, Newcastle upon Tyne NE1 7RU, UK; 2 School of Automation Engineering, University of Electronic Science and Technology of China, Chengdu 610054, China; bin_gao@uestc.edu.cn; 3 Department of Computer and Information Sciences, Northumbria University, Newcastle upon Tyne NE1 8ST, UK; ahmed.bouridane@northumbria.ac.uk; 4 Faculty of Information Engineering, Guangdong University of Technology, Guangzhou 510006, China; yongquanling@gdut.edu.cn; 5 Faculty of Science Agriculture and Engineering, Newcastle University, Singapore 599493, Singapore; cheng.chin@ncl.ac.uk

**Keywords:** adaptive signal processing, blind source separation, sensors signal processing, machine learning, maximization–minimization algorithm, *β*-divergence, matrix deconvolution

## Abstract

This paper presents an unsupervised learning algorithm for sparse nonnegative matrix factor time–frequency deconvolution with optimized fractional β-divergence. The β-divergence is a group of cost functions parametrized by a single parameter β. The Itakura–Saito divergence, Kullback–Leibler divergence and Least Square distance are special cases that correspond to β=0, 1, 2, respectively. This paper presents a generalized algorithm that uses a flexible range of β that includes fractional values. It describes a maximization–minimization (MM) algorithm leading to the development of a fast convergence multiplicative update algorithm with guaranteed convergence. The proposed model operates in the time–frequency domain and decomposes an information-bearing matrix into two-dimensional deconvolution of factor matrices that represent the spectral dictionary and temporal codes. The deconvolution process has been optimized to yield sparse temporal codes through maximizing the likelihood of the observations. The paper also presents a method to estimate the fractional β value. The method is demonstrated on separating audio mixtures recorded from a single channel. The paper shows that the extraction of the spectral dictionary and temporal codes is significantly more efficient by using the proposed algorithm and subsequently leads to better source separation performance. Experimental tests and comparisons with other factorization methods have been conducted to verify its efficacy.

## 1. Introduction

Blind source separation (BSS) [1,2,3,4,5,6,7,8] is an ill-posed problem that cannot be totally solved without some prior information. This entails a certain number of assumptions have to be imposed to render the problem solvable such as channel type (linear [1] versus nonlinear [2]), mutual statistical independence among the sources [3], the number of sources [4], how the sources are mixed (instantaneous [5] versus convolutive [6]), and the location of the sources with respect to the microphones. Several recent solutions have been developed to mitigate some of these constraints. In the work [7], it was previously shown that non-Gaussian stationary process can be approximated as non-stationary Gaussian process which enabled separation involving mixtures of non-Gaussian sources. Of similar concept, a method is proposed for separation by decorrelating multiple non-stationary stochastic sources using a multivariable crosstalk-resistant adaptive noise canceller [8]. In a related method, the problem of speech quality enhancement is tackled using adaptive and non-adaptive filtering algorithms [9]. A two-microphone Gauss–Seidel pseudo affine projection algorithm combined with forward blind source separation is proposed. A higher efficiency in speech enhancement in noisy environment has been attained. The paper [10] proposes rational polynomial functions to replace the original score functions used in standard independent component analysis (ICA). The rational polynomials are derived by the Pade approximant from Taylor series expansion of the original nonlinearities which can be quickly evaluated to enable large-scale multidimensional sets of data characterized by super-Gaussian distribution to be separated within a short period of time. Recently, a bi-variate empirical mode decomposition algorithm combined with complex ICA by entropy bound minimization technique is proposed for convolutive signal separation [11]. In telecommunication problems, neither the direction of arrival (DOA) nor a training sequence is assumed to be available at the receiver. The only assumption is that the transmitted signals satisfy the constant modulus property. In the work [12], a multistage space–time equalizer is proposed to blindly separate signals received by an antenna array from different sources simultaneously. In the algorithm, each stage consists of an adaptive beamformer, a DOA estimator and an equalizer which are jointly optimized using the constant modulus property of the sources. Other than statistical independence and non-Gaussianity, signal separation approach based on second-order statistics of the speech signals using canonical correlation approach [13] has also been proposed. The work [14] considers complex-valued mixing matrix estimation and direction-of-arrival estimation of synchronous orthogonal frequency hopping signals in the underdetermined blind source separation (UBSS). A mixing matrix estimation algorithm is proposed by detecting single source points where only one source contributes its power. While traditional algorithms are usually applied in the ideal sparse environment, the work [15] proposes a solution where multiple input multiple output mixed signals are insufficiently sparse in both time and frequency domains under noisy conditions. The work [16] demonstrates the application of UBSS in addresses the mixing of pipe abrasive debris problem and focuses on the superimposed abrasive debris separation of a radial magnetic field abrasive sensor. Through accurately separating and calculating the morphology and amount of the abrasive debris, the abrasive sensor has provided the system with wear trend and sizes estimation of the wear particles.

In recent years, an alternate class of solutions for BSS based on nonnegative matrix factorization (NMF) [17] has been proposed. Compared to ICA, NMF gives a more part based decomposition and the decomposition is unique under certain conditions, making it unnecessary to impose the constraints in the form of orthogonality and independence [18]. These properties have led to a significant interest in NMF lately for its application in areas of BSS [5,19,20,21,22,23,24], pattern recognition [25], and dimensionality reduction [26]. Multiplicative update-based families of parameterized cost functions such as the Csiszar’s divergences [27,28] were also presented. The NMF is a matrix decomposition technique. Let the data matrix **V** be a nonnegative matrix of dimensions I×J. The aim of NMF is to find two matrices **W** and **H** such that:

(1) V=WH

or in scalar form,

(2) $$V_{i,j}=\underset{k}{\sum }W_{i,k}H_{k,j}$$

where *i* = 1, 2, ..., k=1, 2,…, K, and j=1, 2, …, J. When **W** and **H** are nonnegative matrices of dimensions I×K and K×J, then is usually chosen such that

(3) I×K+K×J≪I×J

A sparseness constraint can be added to the cost function [26,27,28,29,30,31], and this can be achieved by regularization using the *L*_1_-norm leading to Sparse NMF (SNMF). Here, “sparseness” refers to a representational scheme where only a few units (out of a large population) are effectively used to represent typical data vectors. In effect, this implies most units taking values close to zero while only few take significantly non-zero values. Several other types of prior distribution over **W** and **H** can be defined, e.g., it is assumed that the prior of **W** and **H** satisfy the exponential density and the prior for the noise variance is chosen as an inverse gamma density [27]. In the work [28], Gaussian distributions are chosen for both **W** and **H**. The model parameters and hyper parameters are adapted by using the Markov chain Monte Carlo (MCMC) [32]. In all cases, a fully Bayesian treatment is applied to approximate inference for both model parameters and hyper parameters. While these approaches increase the accuracy of matrix factorization, it only works efficiently when a large sample dataset is available. Moreover, it consumes significantly high computational complexity at each iteration to adapt the parameters and its hyper parameters. The NMF with the *β*-divergence has been previously used in music signal processing [33,34]. In our previous paper [35], we investigated *β*-divergence for source separation problem. It was shown that improved performance has been attained over integer-based *β*-divergence. Thus, this motivates research of using *β*-divergence for music signal processing and source separation. However, all of these works fixed β to some constant values within 0–2, and have not presented any method to determine the desired β value. This significantly constrains the performance of matrix factorization and its ability in separating mixed sources. In addition, these works do not consider the issue of sparsity of the temporal codes which would undermine the quality of matrix factorization when the β value is inappropriately chosen. The selection of the β value should consider the sparseness constraint used in the cost function.

Regardless of the cost function and sparseness constraint being used, the standard NMF or SNMF models are only satisfactory for solving source separation provided that the spectral frequencies of the analyzed audio signal do not change over time. However, this is not the case for many realistic signals such as music and speech. As a result, the spectral dictionary obtained via the NMF or SNMF decomposition is not adequate to capture the temporal dependency of the frequency patterns within the signal. To remedy the situation, a pragmatic approach is to work on a more holistic model based on matrix factor deconvolution [21,22,23,24]. In this paper, we work with NMF model extended to two-dimensional time–frequency deconvolution of **W** and **H** where (**W**, **H**) are considered as the matrix factors [22]. Mathematically, this is expressed as

(4) $$V_{i,j}=\overset{K}{\underset{k=1}{\sum }}\overset{\tau_{max}}{\underset{\tau =0}{\sum }}\overset{\phi_{max}}{\underset{\phi =0}{\sum }}W_{i-\phi ,k}^{\tau }H_{k,j-\tau }^{\phi }\;V=\overset{\tau_{max}}{\underset{\tau =0}{\sum }}\overset{\phi_{max}}{\underset{\phi =0}{\sum }}\overset{↓\phi }{W^{\tau }}\overset{\to \tau }{H^{\phi }}$$

where i and i represent the frequency and time index, respectively, k indicates the factor number, τ represents the temporal shift and ϕ is the frequency shift. The terms $\tau_{max}$ and $\phi_{max}$ are the maximum temporal and frequency shift, respectively. With this definition, both $W_{i,k}^{\tau }$ and $H_{k,j}^{\phi }$ have tensorial structures with dimension $I\times K\times \tau_{max}$ and $K\times J\times \phi_{max}$, respectively. Thus, $W_{i,k}^{\tau }$ represents the τth-slice of the kth-spectral basis while $H_{k,j}^{\phi }$ represents the associated ϕth-slice of the kth-temporal code. The downward and rightward arrow signs denote the corresponding shifting direction of each column in $W^{\tau }$ and each row in $H^{\phi }$ by the amount indicated by τ and ϕ, respectively.

Model (4) represents both temporal structure and the pitch change which occur when an instrument plays different notes. In the log-frequency spectrogram, the pitch change corresponds to a displacement on the frequency axis. Where previous NMF methods needed one component to model each note for each instrument, Model (4) represents each instrument compactly by a single time–frequency profile convolved in both time and frequency by a time–pitch weight matrix. This model dramatically decreases the number of components needed to model various instruments and effectively solves the blind single channel source separation problem for certain classes of musical signals. When polyphonic music is modeled by factorizing the magnitude spectrogram with NMF, each instrument is modeled by an instantaneous frequency signature which can vary over time. However, the NMF requires multiple basis functions to represent tones with different pitch values. The two-dimensional time–frequency deconvolution model implicitly solves the problem of grouping notes. Thus, all notes for an instrument is an identical pitch shifted time–frequency signature, Model (4) will give better estimates of these signatures, because more examples of different notes are used to compute each time–frequency signature. In the event when this assumption does not hold, it might still hold in a region of notes for an instrument. Furthermore, the two-dimensional time–frequency deconvolution model can explain the spectral differences between two notes of different pitch by the two-dimensional deconvolution of the time–frequency signature.

The novelty of this paper can be summarized as follows: Firstly, a new algorithm is developed for sparse nonnegative matrix factor time–frequency deconvolution optimized with fractional *β*-divergence. Secondly, the maximization–minimization algorithm is developed to derive the auxiliary cost function which caters for any β value. The paper shows that the optimal β that leads to the desired performance is not necessarily limited to the special cases of integer β but extends to fractional values. Thirdly, it is analytically shown that the convergence of the proposed algorithm is guaranteed under the auxiliary function. Fourthly, a method is proposed to estimate the fractional β within the context of monoaural source separation. Finally, the paper proposes an adaptive method to estimate the sparsity parameter for each of the individual temporal code.

The remainder of the paper is organized as follows: In Section 2, the new algorithm for matrix factor time–frequency deconvolution model with *β*-divergence based on the maximization–minimization algorithmic framework is derived. Real application of blind source separation using the proposed method and comparisons with other matrix factorization methods are presented in Section 3. Finally, Section 4 concludes the paper.

## 2. Background

### 2.1. β-Divergence Cost Function

The NMF problem can be written as the minimization of an objective function:

(5) $$D\left(V|WH\right)=\underset{i,j}{\sum }d_{\beta }\left(V_{i,j}{|\Lambda }_{i,j}\right)$$

The general *β*-divergence [24,31] is defined as:

(6) $$d_{\beta }(y|x)=\{\begin{array}{cc} \frac{y^{\beta }}{\beta (\beta -1)}+\frac{x^{\beta }}{\beta }-\frac{yx^{\beta -1}}{\beta -1}, & \beta \to R/\{0,1\}\\ y(\log y-\log x)+(x-y), & \beta =1\\ \frac{y}{x}-\log\frac{y}{x}-1 & \beta =0 \end{array}$$

when β=2, this matches with the first *β*-divergence and the update algorithm is referred to as the “Least Square” [17]. When we use the second *β*-divergence with β=1, the update algorithm is referred to as the “Kullback–Leibler” [17]. When the third *β*-divergence with β=0 is used, the update algorithm is referred to as the “Itakura–Saito” [33]. These algorithms have their own advantages and disadvantages. If the sources have large dynamic difference in the power, the Itakura–Saito divergence would have better performance than other NMF algorithms. The Least Square and Kullback–Leibler NMFs are more suited when the power of sources are close to other. However, it is difficult to define the difference of power between the sources, and therefore it is difficult to choose the algorithms. In this paper, we present the results to show that the best results are not necessarily limited to the above integer β special cases. The use the fractional *β*-divergence is expected to yield more realistic and optimized results than the previous NMF algorithms. For completeness of presentation, the following section briefly reviews the update function based on the Least Square and Kullback–Leibler criterion.

#### 2.1.1. Least Square Distance

The Least Square NMF algorithm introduced by Lee and Seung [17] defines the *β*-divergence as Least Squares divergence when β=2. First, we consider the least square cost function:

(7) $$\begin{array}{cc} C_{LS}=\frac{1}{2}||V-{\Lambda ||}_{F}^{2} & =\underset{i,j}{\sum }d_{2}\left(V_{i,j}{|\Lambda }_{i,j}\right)\\ & =\frac{1}{2}\underset{i,j}{\sum }{\left(V_{i,j}-\Lambda_{i,j}\right)}^{2} \end{array}$$

Differentiating $C_{LS}$ with respect to $W_{i,k}^{\tau }$ and $H_{k,j}^{\phi }$, and plugging the multiplicative update algorithm for $\theta =\left\{{\left(W_{i,k}^{\tau }\right)}_{I\times K\times \tau_{max}},{\left(H_{k,j}^{\phi }\right)}_{K\times J\times \phi_{max}}\right\}$:

$$\theta \leftarrow \theta \cdot (\frac{{\left[\nabla d_{\beta }\left(y|x\right)\right]}_{-}}{{\left[\nabla d_{\beta }\left(y|x\right)\right]}_{+}})$$

where $\partial d_{\beta }\left(y|x\right)/\partial \theta ={\left[\nabla d_{\beta }\left(y|x\right)\right]}_{+}-{\left[\nabla d_{\beta }\left(y|x\right)\right]}_{-}$, which leads to the following $W^{\tau }$ and $H^{\phi }$ updates:

(8) $$W^{\tau }=W^{\tau }\cdot \frac{\sum_{\phi }\overset{↑\phi \leftarrow \tau \;}{V}\cdot {\overset{\to \tau \;}{H^{\phi }}}^{T}}{\sum_{\phi }\overset{↑\phi \leftarrow \tau \;}{\Lambda }\cdot {\overset{\to \tau \;}{H^{\phi }}}^{T}}$$

(9) $$H^{\phi }=H^{\phi }\cdot \frac{\sum_{\tau }{\overset{↓\phi \;}{W^{\tau }}}^{T}\cdot \overset{↑\phi \leftarrow \tau \;}{V}}{\sum_{\tau }{\overset{↓\phi \;}{W^{\tau }}}^{T}\cdot \overset{↑\phi \leftarrow \tau \;}{\Lambda }}$$

where “A·B” represents element-wise multiplication.

#### 2.1.2. Kullback–Liebler Divergence

When β=1, the *β*-divergence is identical to the Kullback–Leibler divergence. The Kullback–Leibler divergence is expressed as:

(10) $$C_{KL}=\underset{i,j}{\sum }d_{1}\left(V_{i,j}|\Lambda_{i,j}\right)=\underset{i,j}{\sum }V_{i,j}\log\frac{V_{i,j}}{\Lambda_{i,j}}-V_{i,j}+\Lambda_{i,j}$$

By following similar steps as the Least Square, we can derive the update function as follow:

(11) $$W^{\tau }=W^{\tau }\cdot \frac{\sum_{\phi }\left(\frac{\overset{↑\phi \leftarrow \tau \;}{V}}{\Lambda }\right)\cdot {\overset{\to \tau \;}{H^{\phi }}}^{T}}{\sum_{\phi }1\cdot \phi^{T}}$$

(12) $$H^{\phi }=H^{\phi }\cdot \frac{\sum_{\tau }{\overset{↓\phi \;}{W^{\tau }}}^{T}\cdot \left(\frac{\overset{↑\phi \leftarrow \tau \;}{V}}{\Lambda }\right)}{\sum_{\tau }{\overset{↓\phi \;}{W^{\tau }}}^{T}\cdot 1}$$

where “$\frac{A}{B}\;$” represents element-wise division and “**1**” is a column vector of unit elements.

### 2.2. Auxiliary Cost Function of Fractional β-Divergence for Matrix Factors Time–Frequency Deconvolution

In this subsection, we introduce the cost function for the fractional β-divergence matrix factors time–frequency deconvolution model. The algorithm allows the user to choose a fractional *β* value instead of using the previous NMF algorithms which constrain *β* to special cases of integer value. After the derivation, this paper shows the steps on how the update function of the fractional *β*-divergence is obtained for the parameters. Firstly, the first derivative of $d_{\beta }\left(y|x\right)$ are given by

(13) $$d_{\beta }^{\prime \left(y|x\right)}=y^{\beta -2}\left(y-x\right)$$

This shows that y is continuous in β and thus the second derivative of $d_{\beta }\left(y|x\right)$ is given by

(14) $$d_{\beta }^{\prime \prime \left(y|x\right)}=y^{\beta -3}\left[\left(\beta -1\right)y-\left(\beta -2\right)x\right]$$

The second derivative shows that the *β*-divergence is convex for y in β∈[1,2]. Outside of this range, $d_{\beta }\left(y|x\right)$ can be expressed as:

(15) $$d_{\beta }\left(y|x\right)=\overset{ˇ}{d}\left(y|x\right)+\hat{d}\left(y|x\right)+\bar{d}\left(x\right)$$

where $\overset{ˇ}{d}\left(y|x\right)$ is a convex function of y, $\hat{d}\left(y|x\right)$ is a concave function of *y*, and $\bar{d}\left(x\right)$ is a constant of *y*. Table 1 shows the various functions for $\overset{ˇ}{d}\left(y|x\right)$, $\hat{d}\left(y|x\right)$ and $\bar{d}\left(x\right)$. The problem we want to tackle is to minimize the following function with respect to $\theta =\left\{{\left(W_{i,k}^{\tau }\right)}_{I\times K\times \tau_{max}},{\left(H_{k,j}^{\phi }\right)}_{K\times J\times \phi_{max}}\right\}$ where β can assume fractional number:

(16) $$\begin{array}{ll} G\left(\theta \right) & =\underset{i,j}{\sum }d_{\beta }\left(V_{i,j}|\sum_{k=1}^{K}\sum_{\tau =0}^{\tau_{max}}\sum_{\phi =0}^{\phi_{max}}W_{i-\phi ,k}^{\tau }H_{k,j-\tau }^{\phi }\right)\\ & =\frac{1}{\beta \left(\beta -1\right)}\underset{i,j}{\sum }V_{i,j}^{\beta }+\underset{i,j}{\sum }\underset{G_{1}\left(\beta \right)}{\underbrace{\frac{1}{\beta }{\left(\underset{k,\tau \phi }{\sum }W_{i-\phi ,k}^{\tau }H_{k,j-\tau }^{\phi }\right)}^{\beta }}}+\underset{i,j}{\sum }V_{i,j}\underset{G_{2}\left(\beta \right)}{\underbrace{{\left(-\frac{1}{\beta -1}\underset{k,\tau \phi }{\sum }W_{i-\phi ,k}^{\tau }H_{k,j-\tau }^{\phi }\right)}^{\beta -1}}} \end{array}$$

In Equation (16), $G_{1}\left(\beta \right)$ is convex for β≥1 and concave for β<1, and $G_{2}\left(\beta \right)$ is convex for β≤2 and concave for β>2. Thus, there is a need to alleviate this problem by decomposing the above function into several terms to be either convex or concave depending on the value of β and use the appropriate inequalities to build an auxiliary function.

**Lemma** **1.** *For the case of β≥1, we have*

(17) $$\frac{1}{\beta }{\left(\underset{k,\tau \phi }{\sum }W_{i-\phi ,k}^{\tau }H_{k,j-\tau }^{\phi }\right)}^{\beta }\leq \frac{1}{\beta }\underset{k,\tau \phi }{\sum }\omega_{i,j,k,\tau ,\phi }{\left(\frac{W_{i-\phi ,k}^{\tau }H_{k,j-\tau }^{\phi }}{\omega_{i,j,k,\tau ,\phi }}\right)}^{\beta }=P_{i,j}^{\left(\beta \right)}$$

*where $\omega_{i,j,k,\tau ,\phi }\geq 0$ for all k,τ,ϕ and $\sum_{k,\tau \phi }\omega_{i,j,k,\tau ,\phi }=1$. The equality holds when*

(18) $$\omega_{i,j,k,\tau ,\phi }=\frac{W_{i-\phi ,k}^{\tau }H_{k,j-\tau }^{\phi }}{\sum_{k^{\prime },\tau^{\prime },\phi^{\prime }}W_{i-\phi^{\prime },k^{\prime }}^{\tau^{\prime }}H_{k^{\prime },j-\tau^{\prime }}^{\phi^{\prime }}}$$

**Proof.**  Let f:R→R be a convex function. If $\alpha_{k}\left(k=1,\;2,\ldots ,K\right)$ satisfies $\forall k,\alpha_{k}>0$ and $\sum_{k}\alpha_{k}=1$, then for any $x_{k}\left(k=1,\;2,\ldots ,K\right)\in R$,

$$f\left(\underset{k}{\sum }x_{k}\right)\leq \underset{k}{\sum }\alpha_{k}f\left(\frac{x_{k}}{\alpha_{k}}\right)$$

and with equality holds if and only if $\alpha_{k}=x_{k}/\sum_{k}x_{k}$. Substituting $f\left(\cdot \right)=\frac{1}{\beta }{\left(\cdot \right)}^{\beta }$ with β≥1, $x_{k}=W_{i-\phi ,k}^{\tau }H_{k,j-\tau }^{\phi }$ and $\alpha_{k}=\omega_{i,j,k,\tau ,\phi }$ yields Equation (16). ☐

**Lemma** **2.** *For the case of β<1, we have*

(19) $$\frac{1}{\beta }{\left(\underset{k,\tau \phi }{\sum }W_{i-\phi ,k}^{\tau }H_{k,j-\tau }^{\phi }\right)}^{\beta }\leq \Lambda_{i,j}^{\beta -1}\left(\underset{k,\tau \phi }{\sum }W_{i-\phi ,k}^{\tau }H_{k,j-\tau }^{\phi }-\Lambda_{i,j}\right)+\frac{\Lambda_{i,j}^{\beta }}{\beta }=Q_{i,j}^{\left(\beta \right)}$$

*The equality holds when*

(20) $$\Lambda_{i,j}=\underset{k,\tau \phi }{\sum }W_{i-\phi ,k}^{\tau }H_{k,j-\tau }^{\phi }$$

**Proof.**  Let f:R→R be a continuously differentiable and concave function. Then, for any point z,

$$f\left(x\right)\leq f^{\prime }\left(x\right)\left(x-z\right)+f\left(z\right)$$

and with equality holds if and only if x=z. Substituting $f\left(\cdot \right)=\frac{1}{\beta }{\left(\cdot \right)}^{\beta }$ with β<1, $x=\sum_{k,\tau \phi }W_{i-\phi ,k}^{\tau }H_{k,j-\tau }^{\phi }$ and $z=\Lambda_{i,j}$ yields Equation (17). ☐

Using Lemmas 1 and 2, we may proceed with the following analysis. When β<1, we use $Q_{i,j}^{\left(\beta \right)}$ instead of $G_{1}\left(\beta \right)$ and $V_{i,j}P_{i,j}^{\left(\beta -1\right)}$ instead of $G_{2}\left(\beta \right)$, then the cost function becomes a convex function. Let us denote $G^{+}\left(\theta |\hat{\theta }\right)$ as an auxiliary function for and $\hat{\theta }=\left\{{\left(\omega_{i,j,k,\tau ,\phi }\right)}_{I\times J\times K\times \tau_{max}\times \phi_{max}},{\left(\Lambda_{i,j}\right)}_{I\times J}\right\}$ as the auxiliary variables. For $G^{+}\left(\theta |\hat{\theta }\right)$ to qualify as auxiliary function, it must satisfy $G\left(\theta \right)=\underset{\hat{\theta }}{\min}G^{+}\left(\theta |\hat{\theta }\right)$. Thus, the cost function can be shown to be bounded by the auxiliary function $G^{+}\left(\theta |\hat{\theta }\right)$:

(21) $$G\left(\theta \right)\leq G^{+}\left(\theta |\hat{\theta }\right)=\underset{i,j}{\sum }\frac{V_{i,j}^{\beta }}{\beta \left(\beta -1\right)}+Q_{i,j}^{\left(\beta \right)}-V_{i,j}P_{i,j}^{\left(\beta -1\right)}$$

when 1≤β≤2, we use $P_{i,j}^{\left(\beta \right)}$ instead of $G_{1}\left(\beta \right)$ and $V_{i,j}P_{i,j}^{\left(\beta -1\right)}$ instead of $G_{2}\left(\beta \right)$, then the cost function becomes a convex function and is bounded by the auxiliary function $G^{+}\left(\theta |\hat{\theta }\right)$:

(22) $$G\left(\theta \right)\leq G^{+}\left(\theta |\hat{\theta }\right)=\underset{i,j}{\sum }\frac{V_{i,j}^{\beta }}{\beta \left(\beta -1\right)}+P_{i,j}^{\left(\beta \right)}-V_{i,j}P_{i,j}^{\left(\beta -1\right)}$$

Finally, when β>2, we use $P_{i,j}^{\left(\beta \right)}$ instead of $G_{1}\left(\beta \right)$ and $V_{i,j}Q_{i,j}^{\left(\beta -1\right)}$ instead of $G_{2}\left(\beta \right)$, then the cost function is bounded by

(23) $$G\left(\theta \right)\leq G^{+}\left(\theta |\hat{\theta }\right)=\underset{i,j}{\sum }\frac{V_{i,j}^{\beta }}{\beta \left(\beta -1\right)}+P_{i,j}^{\left(\beta \right)}-V_{i,j}Q_{i,j}^{\left(\beta -1\right)}$$

From above, we can conclude that

(24) $$G\left(\theta \right)\leq G^{+}\left(\theta |\hat{\theta }\right)=\underset{i,j}{\sum }\frac{V_{i,j}^{\beta }}{\beta \left(\beta -1\right)}+\{\begin{array}{cc} Q_{i,j}^{\left(\beta \right)}-V_{i,j}P_{i,j}^{\left(\beta -1\right)}, & \left(\beta <1\right)\\ P_{i,j}^{\left(\beta \right)}-V_{i,j}P_{i,j}^{\left(\beta -1\right)}\;, & \left(1\leq \beta \leq 2\right)\\ P_{i,j}^{\left(\beta \right)}-V_{i,j}Q_{i,j}^{\left(\beta -1\right)}\;, & \left(\beta >2\right) \end{array}$$

The equality holds when $\hat{\theta }$ satisfies Equations (18) and (20). The above function yields three different sub-functions which depend on the β value. In different β range, we use different cost function in the algorithm. This allows the user to choose the optimal β value to separate the mixture and caters for more flexibility than the previous algorithms.

### 2.3. Auxiliary Update Function of “Fractional” β-Divergence

To minimize $G^{+}\left(\theta |\hat{\theta }\right)$, we formulate the derivative of $G^{+}\left(\theta |\hat{\theta }\right)$ with respect to θ. First, we consider the derivative for $W_{i,k}^{\tau }$:

(25) $$\frac{\partial G^{+}\left(\theta |\hat{\theta }\right)}{\partial W_{i,k}^{\tau }}=V_{w}-W_{w}$$

where

(26) $$V_{w}=\{\begin{array}{ll} \underset{j,\phi }{\sum }\Lambda_{i,j}^{\beta -1}H_{k,j-\tau }^{\phi }, & \left(\beta <1\right)\\ {\left(W_{i,k}^{\tau }\right)}^{\beta -1}\underset{j,\phi }{\sum }\omega_{i+\phi ,j,k,\tau ,\phi }^{1-\beta }{\left(H_{k,j-\tau }^{\phi }\right)}^{\beta }, & \left(\beta \geq 1\right) \end{array}$$

(27) $$W_{w}=\{\begin{array}{cc} {\left(W_{i,k}^{\tau }\right)}^{\beta -2}\underset{j,\phi }{\sum }V_{i+\phi ,j}\omega_{i+\phi ,j,k,\tau ,\phi }^{2-\beta }{\left(H_{k,j-\tau }^{\phi }\right)}^{\beta -1} & \left(\beta \leq 2\right)\\ \underset{j,\phi }{\sum }V_{i+\phi ,j}\Lambda_{i+\phi ,j}^{\beta -2}H_{k,j-\tau }^{\phi }\;, & \left(\beta >2\right) \end{array}$$

The second derivative of $G^{+}\left(\theta |\hat{\theta }\right)$ with respect to $W_{i,k}^{\tau }$ in then expressed as:

(28) $$\frac{\partial^{2}G^{+}\left(\theta |\hat{\theta }\right)}{\partial W_{i,k}^{\tau }\partial W_{i^{\prime },k^{\prime }}^{\tau^{\prime }}}=(V_{w}{}^{\prime }-W_{w}{}^{\prime })\delta_{i,i^{\prime }}\delta_{k,k^{\prime }}\delta_{\tau ,\tau^{\prime }}$$

where $\delta_{i,j}=1$ if i=j and $\delta_{i,j}=0$ if i≠j, and

(29) $$V_{w}^{\prime }=\{\begin{array}{cc} 0, & \left(\beta <1\right)\\ \left(\beta -1\right){\left(W_{i-\phi ,k}^{\tau }\right)}^{\beta -2}\underset{j,\phi }{\sum }\omega_{i+\phi ,j,k,\tau ,\phi }^{1-\beta }{\left(H_{k,j-\tau }^{\phi }\right)}^{\beta }\;,\;\;\left(\beta \geq 1\right) & \left(\beta \geq 1\right) \end{array}$$

(30) $$W_{w}^{\prime }=\{\begin{array}{cc} \left(\beta -2\right){\left(W_{i,k}^{\tau }\right)}^{\beta -3}\underset{j,\phi }{\sum }V_{i+\phi ,j}\omega_{i+\phi ,j,k,\tau ,\phi }^{2-\beta }{\left(H_{k,j-\tau }^{\phi }\right)}^{\beta -1}, & \left(\beta \leq 2\right)\\ 0, & \left(\beta >2\right) \end{array}$$

We can see $G\left(\theta |\hat{\theta }\right)$ is a convex function in $W_{i,k}^{\tau }$, so by setting $\frac{\partial G\left(\theta |\hat{\theta }\right)}{\partial W_{i,k}}$ to 0, we can then express the update function for $W_{i,k}^{\tau }$ as:

(31) $$W_{i,k}^{\tau }=\{\begin{array}{cc} {\left(\frac{\sum_{j,\phi }V_{i+\phi ,j}\;\omega_{i+\phi ,j,k,\tau ,\phi }^{2-\beta }{\left(H_{k,j-\tau }^{\phi }\right)}^{\beta -1}}{\sum_{j,\phi }\Lambda_{i,j}^{\beta -1}H_{k,j-\tau }^{\phi }}\right)}^{\frac{1}{2-\beta }}\;, & \left(\beta <1\right)\\ \frac{\sum_{j,\phi }V_{i+\phi ,j}\;\omega_{i+\phi ,j,k,\tau ,\phi }^{2-\beta }{\left(H_{k,j-\tau }^{\phi }\right)}^{\beta -1}}{\sum_{j,\phi }\omega_{i+\phi ,j,k,\tau ,\phi }^{1-\beta }{\left(H_{k,j-\tau }^{\phi }\right)}^{\beta }}\;, & \left(1\leq \beta \leq 2\right)\\ {\left(\frac{\sum_{j,\phi }V_{i+\phi ,j}\;\Lambda_{i+\phi ,j}^{\beta -2}\;H_{k,j-\tau }^{\phi }}{\sum_{j,\phi }\omega_{i+\phi ,j,k,\tau ,\phi }^{1-\beta }{\left(H_{k,j-\tau }^{\phi }\right)}^{\beta }}\right)}^{\frac{1}{\beta -1}}\;, & \left(\beta >2\right) \end{array}$$

We next consider the auxiliary variables $\hat{\theta }$. Since both Equations (17) and (18) minimize $G^{+}\left(\theta |\hat{\theta }\right)$ with respect to $\hat{\theta }$, substituting these into Equation (30) gives the following update rule:

(32) $$W_{i,k}^{\tau }=W_{i,k}^{\tau }{\left(\frac{\sum_{j,\phi }V_{i+\phi ,j}\;\Lambda_{i+\phi ,j}^{\beta -2}\;H_{k,j-\tau }^{\phi }}{\sum_{j,\phi }\Lambda_{i+\phi ,j}^{\beta -1}\;H_{k,j-\tau }^{\phi }}\right)}^{\delta \left(\beta \right)}$$

where

(33) $$\delta \left(\beta \right)=\{\begin{array}{cc} \frac{1}{2-\beta }, & \left(\beta <1\right)\\ \;1, & \left(1\leq \beta \leq 2\right)\\ \frac{1}{\beta -1}, & \left(\beta >2\right) \end{array}$$

The above can be written in the matrix form:

(34) $$W^{\tau }=W^{\tau }\cdot {\left[\frac{\sum_{\phi }\left(\overset{↑\phi }{V}\cdot \overset{↑\phi }{\Lambda^{\left(\beta -2\right)}}\right){\overset{\to \tau \;}{H^{\phi }}}^{T}}{\sum_{\phi }\overset{↑\phi \;}{\Lambda^{\left(\beta -1\right)}}{\overset{\to \tau }{H^{\phi }}}^{T}}\right]}^{\delta \left(\beta \right)}$$

Similarly, for $H_{k,j}^{\phi }$ update function, first we have

(35) $$\frac{\partial G^{+}\left(\theta |\hat{\theta }\right)}{\partial H_{k,j}^{\phi }}=V_{H}-W_{H}$$

where

(36) $$V_{H}=\{\begin{array}{ll} \underset{i,\tau }{\sum }\Lambda_{i,j+\tau }^{\beta -1}\;W_{i-\phi ,k}^{\tau }\;, & \left(\beta <1\right)\\ {\left(H_{k,j}^{\phi }\right)}^{\beta -1}\underset{i,\tau }{\sum }\omega_{i,j+\tau ,k,\tau ,\phi }^{1-\beta }{\left(W_{i-\phi ,k}^{\tau }\right)}^{\beta }\;, & \left(\beta \geq 1\right) \end{array}$$

(37) $$W_{H}=\{\begin{array}{cc} {\left(H_{k,j}^{\phi }\right)}^{\beta -2}\underset{i,\tau }{\sum }V_{i,j+\tau }\;\omega_{i,j+\tau ,k,\tau ,\phi }^{2-\beta }{\left(W_{i-\phi ,k}^{\tau }\right)}^{\beta -1}\;, & \left(\beta \leq 2\right)\\ \underset{i,\tau }{\sum }V_{i,j+\tau }\Lambda_{i,j+\tau }^{\beta -2}W_{i-\phi ,k}^{\tau }\;, & \left(\beta >2\right) \end{array}$$

From Equations (17)–(31), we can minimize the cost-function by setting $\frac{\partial G_{s}\left(\theta |\hat{\theta }\right)}{\partial H_{k,j}^{\varphi }}=0$ and obtain $H_{k,j}^{\phi }$ as:

(38) $$H_{k,j}^{\phi }=\{\begin{array}{cc} {\left(\frac{\sum_{i,\tau }V_{i,j+\tau }\;\omega_{i,j+\tau ,k,\tau ,\phi }^{2-\beta }{\left(W_{i-\phi ,k}^{\tau }\right)}^{\beta -1}}{\sum_{i,\tau }\Lambda_{i,j+\tau }^{\beta -1}\;W_{i-\phi ,k}^{\tau }}\right)}^{\frac{1}{2-\beta }} & \left(\beta <1\right)\\ \frac{\sum_{i,\tau }V_{i,j+\tau }\;\omega_{i,j+\tau ,k,\tau ,\phi }^{2-\beta }{\left(W_{i-\phi ,k}^{\tau }\right)}^{\beta -1}}{\sum_{i,\tau }\omega_{i,j+\tau ,k,\tau ,\phi }^{1-\beta }{\left(W_{i-\phi ,k}^{\tau }\right)}^{\beta }} & \left(1\leq \beta \leq 2\right)\\ {\left(\frac{\sum_{i,\tau }V_{i,j+\tau }\Lambda_{i,j+\tau }^{\beta -2}W_{i-\phi ,k}^{\tau }}{\sum_{i,\tau }\omega_{i,j+\tau ,k,\tau ,\phi }^{1-\beta }{\left(W_{i-\phi ,k}^{\tau }\right)}^{\beta }}\right)}^{\frac{1}{\beta -1}}, & \left(\beta >2\right) \end{array}$$

Again, since both Equations (19) and (20) minimizes $G^{+}\left(\theta |\hat{\theta }\right)$ with respect to $\hat{\theta }$, substituting these into Equation (38) gives the following update rule for $H_{k,j}^{\phi }$:

(39) $$H_{k,j}^{\phi }=H_{k,j}^{\phi }{\left(\frac{\sum_{i,\tau }V_{i,j+\tau }\;\Lambda_{i,j+\tau }^{\beta -2}\;W_{i-\phi ,k}^{\tau }}{\sum_{i,\tau }\Lambda_{i,j+\tau }^{\beta -1}\;W_{i-\phi ,k}^{\tau }}\right)}^{\delta \left(\beta \right)}$$

In matrix form, the above can be written as

(40) $$H^{\phi }=H^{\phi }\cdot {\left[\frac{\sum_{\tau }{\overset{↓\phi \;}{W^{\tau }}}^{T}\left(\overset{\leftarrow \tau \;}{V}\cdot \overset{\leftarrow \tau \;}{\Lambda^{\left(\beta -2\right)}}\right)}{\sum_{\tau }{\overset{↓\phi \;}{W^{\tau }}}^{T}\overset{\leftarrow \tau \;}{\Lambda^{\left(\beta -1\right)}}}\right]}^{\delta \left(\beta \right)}$$

### 2.4. Sparsity-Aware Optimization

The cost-function in Equation (21) can be augmented with a regularization term to render sparsity to the solution. We can define a prior on H as an exponential distribution with independent decay parameters, namely,

(41) $$p\left(H|\lambda \right)=\underset{\phi }{\prod }p\left(H^{\phi }|\lambda^{\phi }\right)=\underset{\phi }{\prod }\underset{k}{\prod }\underset{j}{\prod }p\left(H_{k,j}^{\phi }|\lambda_{k,j}^{\phi }\right)$$

where $p\left(H_{k,j}^{\phi }|\lambda_{k,j}^{\phi }\right)=\prod_{\phi }\prod_{k}\prod_{j}\lambda_{k,j}^{\phi }\exp\left(-\lambda_{k,j}^{\phi }H_{k,j}^{\phi }\right)$. The negative log prior on H is defined as $-\log p\left(H|\lambda \right)=f\left(H\right)=\sum_{\phi ,k,j}\left(\lambda_{k,j}^{\phi }H_{k,j}^{\phi }-\log\lambda_{k,j}^{\phi }\right)$. It is worth pointing out that *each individual element* in H is constrained to an exponential distribution with independent decay parameter $\lambda_{k,j}^{\phi }$ so that each element in H can be driven to be optimally sparse in the $L_{1}$-norm. Other forms of sparseness exist^19^ but the proposed $L_{1}$-norm is computationally favourable. First, we define $G_{s}\left(\theta \right)$ and $G_{s}\left(\theta |\hat{\theta }\right)$ as follow:

(42) $$\begin{array}{ll} G_{s}\left(\theta \right) & ≜G\left(\theta \right)+\alpha f\left(H\right)\\ & \leq G^{+}\left(\theta |\hat{\theta }\right)+\alpha f\left(H\right)\\ & =G_{s}\left(\theta |\hat{\theta }\right) \end{array}$$

where α is the regularization constant. To avoid the scaling misbehavior when incorporating the sparseness for H, we reformulate the cost function to work with normalized matrix for $W^{\tau }$ i.e.,

(43) $${\bar{W}}_{i,k}^{\tau }=\frac{W_{i,k}^{\tau }}{\sqrt{\sum_{\tau ,i}{\left(W_{i,k}^{\tau }\right)}^{2}}}=\frac{W_{i,k}^{\tau }}{||W_{k}{\left|\right|}_{2}}$$

and

(44) $${\bar{\Lambda }}_{i,j}=\underset{k,\tau ,\phi }{\sum }{\bar{W}}_{i-\phi ,k}^{\tau }\;H_{k,j-\tau }^{\phi }$$

Thus, the cost function takes the following form:

(45) $$\begin{array}{ll} G_{s}\left(\theta \right) & \leq G_{s}\left(\theta |\hat{\theta }\right)\\ & =\underset{i,j}{\sum }\frac{V_{i,j}^{\beta }}{\beta \left(\beta -1\right)}+\alpha \underset{\phi ,k,j}{\sum }\left(\lambda_{k,j}^{\phi }H_{k,j}^{\phi }-\log\lambda_{k,j}^{\phi }\right)+\{\begin{array}{cc} {\bar{Q}}_{i,j}^{\left(\beta \right)}-V_{i,j}{\bar{P}}_{i,j}^{\left(\beta -1\right)}\;, & \left(\beta <1\right)\\ {\bar{P}}_{i,j}^{\left(\beta \right)}-V_{i,j}{\bar{P}}_{i,j}^{\left(\beta -1\right)\;,} & \left(1\leq \beta \leq 2\right)\\ {\bar{P}}_{i,j}^{\left(\beta \right)}-V_{i,j}{\bar{Q}}_{i,j}^{\left(\beta -1\right)\;,} & \left(\beta >2\right) \end{array} \end{array}$$

where

$${\bar{Q}}_{i,j}^{\left(\beta \right)}={\bar{\Lambda }}_{i,j}^{\beta -1}\left(\underset{k,\tau \phi }{\sum }{\bar{W}}_{i-\phi ,k}^{\tau }H_{k,j-\tau }^{\phi }-{\bar{\Lambda }}_{i,j}\right)+\frac{{\bar{\Lambda }}_{i,j}^{\beta -1}}{\beta }$$

and

$${\bar{P}}_{i,j}^{\left(\beta \right)}=\frac{1}{\beta }\underset{k,\tau ,\phi }{\sum }\omega_{i,j,k,\tau ,\phi }{\left(\frac{{\bar{W}}_{i-\phi ,k}^{\tau }H_{k,j-\tau }^{\phi }}{\omega_{i,j,k,\tau ,\phi }}\right)}^{\beta }$$

To obtain $\lambda_{k,j}^{\phi }$, we minimize the cost function with respect $\lambda_{k,j}^{\phi }$ and set it to zero which results:

(46) $$\lambda_{k,j}^{\phi }=\frac{1}{H_{k,j}^{\phi }}$$

provided that $H_{k,j}^{\phi }\neq 0$. However, it has been observed in many cases that optimizing the factor matrices with *β*-divergence and the sparseness in Equation (46) increases the likelihood for some $H_{k,j}^{\phi }$ to converge very close to zero, thus leading to numerical divergence when dividing by zero. Other practices introduced a small constant to $H_{k,j}^{\phi }$ to prevent direct division by zero. Unfortunately, such approach is identical to constant sparsity and no longer preserves the $L_{1}$-norm optimal solution. In this paper, we adopt the maximum likelihood approach to formulating the adaptive estimation of sparsity parameter $\lambda_{k,j}^{\phi }$. Considering the following maximum likelihood criterion [31,36]:

(47) $$\lambda^{ML}=\arg\;\underset{\lambda }{\max}\ln p(v|\lambda ,\overset{ˇ}{W})$$

where $\ln p(v|\lambda ,\overset{ˇ}{W})$ is the log-likelihood conditional probability of the observations given $\overset{ˇ}{W}$ and λ. By using the Jensen’s inequality, for any distribution Q(h), the log-likelihood function satisfies the following:

(48) $$\begin{array}{ll} \lambda^{ML} & =\arg\;\underset{\lambda }{\max}\int^{\text{​}}Q\left(h\right)\ln p\left(v,h|\lambda ,\overset{ˇ}{W}\right)dh\\ & =\arg\;\underset{\lambda }{\max}\int^{\text{​}}Q\left(h\right)(\ln p\left(v|h,\overset{ˇ}{W}\right)+\ln p\left(h|\lambda \right))dh\\ & =\arg\;\underset{\lambda }{\max}\int^{\text{​}}Q\left(h\right)\ln p\left(h|\lambda \right)dh \end{array}$$

Since each element of H is constrained to be exponential distributed with independent decay parameters, Equation (48) becomes:

(49) $$\lambda_{g}^{ML}=\arg\;\underset{\lambda }{\max}\int Q\left(h\right)\left(\ln\lambda_{g}-\lambda_{g}h_{g}\right)dh$$

Thus, we have

(50) $$\lambda_{g}^{ML}=\frac{1}{\int h_{g}Q\left(h\right)dh}\;$$

One can easily check that the distribution that maximizes the maximum likelihood is given by $Q\left(h\right)=p\left(h|v,\lambda ,\;\overset{ˇ}{W}\right)=p\left(v|h,\lambda ,\;\overset{ˇ}{W}\right)p\left(h|\lambda \right)/p\left(v|\lambda ,\;\overset{ˇ}{W}\right)$ which is the posterior distribution of h and $p\left(v|h,\lambda ,\;\overset{ˇ}{W}\right)$ is the log-likelihood function of the observation which is usually expressed by a Gaussian density function with mean centered at $\sum_{k,\tau ,\phi }{\bar{W}}_{i-\phi ,k}^{\tau }\;H_{k,j-\tau }^{\phi }$. However, as $H_{k,j}^{\phi }$ is directly acquired from the original code matrix $H_{k,j}^{0}$, we can simply work with $\tau_{max}=0$. This allows us to express the log-likelihood function of the posterior distribution of h up to a constant as

(51) $$\begin{array}{ll} \ln p\left(h|v,\lambda ,\;\overset{ˇ}{W}\right) & \dot{=}\ln p\left(v|h,\lambda ,\;\overset{ˇ}{W}\right)+\ln p\left(h|\lambda \right)\\ & \dot{=}\frac{1}{2}||vec\left(V\right)-\underset{\phi }{\sum }\left(I⨂\overset{↓\phi \;}{\bar{W}}\right)vec\left(H^{\phi }\right){\left|\right|}_{F}^{2}+\alpha \underset{\phi }{\sum }\left\{{\left(\lambda^{\phi }\right)}^{T}vec\left(H^{\phi }\right)-{\left(\log\lambda^{\phi }\right)}^{T}1\right\}\\ & =F\left(H,\lambda \right) \end{array}$$

where “$\dot{=}$” denotes equality up to a constant, “⨂“ is the Kronecker product, **1** is vector contains unit elements, I is the identity matrix, α assumes the role of a regularization constant to balance the cost function fit and smoothness of H. For ease of presentation, we simplify the above terms as v=vec(V), $\overset{ˇ}{W}=\left[\begin{array}{ccc} I⨂\overset{↓0\;}{\bar{W}} & \ldots & I⨂\overset{↓\phi_{max}\;}{\bar{W}} \end{array}\right]$, $h=\left\{h_{g}\right\}={\left[\begin{array}{ccc} vec{\left(H^{0}\right)}^{T} & \cdots & vec{\left(H^{\phi_{max}}\right)}^{T} \end{array}\right]}^{T}$, $\lambda =\left\{\lambda_{g}\right\}={\left[\begin{array}{ccc} \lambda^{0}{}^{T} & \ldots & \lambda^{\phi_{max}}{}^{T} \end{array}\right]}^{T}$ which enables us to rewrite Equation (46) as

(52) $$F\left(H,\lambda \right)=\frac{1}{2}||v-\overset{ˇ}{W}h{\left|\right|}_{F}^{2}+\alpha \left(\lambda^{T}h-{\left(\log\lambda \right)}^{T}1\right)$$

For ease of analysis, Q(h) is represented using Gibbs distribution as $Q\left(h\right)=\frac{1}{Z}\exp\left(-F\left(h\right)\right)$ where Z=∫exp(−F(h))dh. Let P represents the index set of inactive code i.e., $P=\left\{\phi ,k,j|H_{k,j}^{\phi }=0\right\}$ and M the index set of active code i.e., $M=\left\{\phi ,k,j|H_{k,j}^{\phi }\neq 0\right\}$. Thus, Q(h) can be factorized as

(53) $$\begin{array}{ll} Q\left(h\right) & =\frac{1}{Z}\exp\left(-F\left(h,\lambda \right)\right)\\ & \approx \frac{1}{Z_{P}}\exp\left(-F\left(h_{P},\lambda_{P}\right)\right)\frac{1}{Z_{M}}\exp\left(-F\left(h_{M},\lambda_{M}\right)\right)\\ & =Q_{P}\left(h_{P}\right)Q_{M}\left(h_{M}\right) \end{array}$$

Since $h_{M}$ corresponds to the original non-zero value of h, it then follows that $Q_{M}\left(h_{M}\right)$ is not of interest to us. We are only interested in $h_{P}$ and therefore, to characterize $Q_{P}\left(h_{P}\right)$, we need to allow some positive deviation to $h_{P}$. A suitable distribution is to use the factorized exponential distribution given by

(54) $${\hat{Q}}_{P}\left(h_{P}\geq 0\right)=\underset{p\in P}{\prod }\frac{1}{u_{p}}\exp\left(-\frac{h_{p}}{u_{p}}\right)$$

as the approximate distribution. The variational parameters $u=\left\{u_{p}\right\}$ are determined by minimizing the Kullback–Leibler divergence between true $Q_{P}$ and approximate ${\hat{Q}}_{P}$:

(55) $$\begin{array}{ll} u & =\arg\;\underset{u}{\min}\int^{\text{​}}{\hat{Q}}_{P}\left(h_{P}\right)\ln\frac{{\hat{Q}}_{P}\left(h_{P}\right)}{Q_{P}\left(h_{P}\right)}dh_{P}\\ & =\arg\;\underset{u}{\min}\int^{\text{​}}{\hat{Q}}_{P}\left(h_{P}\right)\left\{\ln{\hat{Q}}_{P}\left(h_{P}\right)-Q_{P}\left(h_{P}\right)\right\}dh_{P} \end{array}$$

which leads to the following optimization:

(56) $$\underset{u_{p}}{\min}b_{P}^{T}u+\frac{1}{2}\;u^{T}Cu-\underset{p\in P}{\sum }\ln u_{p}$$

where $b_{P}={\left(Ch-{\overset{ˇ}{W}}^{T}v+\lambda \right)}_{P}$ and $C=C_{P}+diag\left(C_{P}\right)$ with $C={\overset{ˇ}{W}}^{T}\overset{ˇ}{W}$, $C_{P}={\overset{ˇ}{W}}_{P}^{T}{\overset{ˇ}{W}}_{P}$. Solving Equation (56) for $u_{p}$ leads to the following update:

(57) $$u_{p}\leftarrow u_{p}\frac{-b_{p}+\sqrt{b_{p}^{2}+4\frac{{\left(Cu\right)}_{p}}{u_{p}}}}{2{\left(Cu\right)}_{p}}$$

Once $u_{p}$ is obtained and re-arranged to the original form $u_{k,j}^{\phi }$, the final update for $\lambda_{k,j}^{\phi }$ takes the form of:

(58) $$\lambda_{k,j}^{\phi }=\frac{1}{H_{k,j}^{\phi }+\delta_{k,j}^{\phi }}$$

where

(59) $$\delta_{k,j}^{\phi }=\{\begin{array}{c} \;0\;\mathrm{if}\;H_{k,j}^{\phi }\neq 0\;\\ u_{k,j}^{\phi }\;\mathrm{if}\;H_{k,j}^{\phi }=0 \end{array}$$

Equipped with above, we obtain the multiplicative update for the normalized W as

(60) $$W^{\tau }={\bar{W}}^{\tau }\cdot {\left[\frac{\sum_{\phi }\left(\overset{↑\phi \;}{V}\cdot \overset{↑\phi \;}{{\bar{\Lambda }}^{\left(\beta -2\right)}}\right){\overset{\to \tau \;}{H^{\phi }}}^{T}+{\bar{W}}^{\tau }diag\left(\sum_{\tau }1\left(\left(\overset{↑\phi \;}{{\bar{\Lambda }}^{\left(\beta -1\right)}}{\overset{\to \tau \;}{H^{\phi }}}^{T}\right)\cdot {\bar{W}}^{\tau }\right)\right)}{\sum_{\phi }\overset{↑\phi \;}{{\bar{\Lambda }}^{\left(\beta -1\right)}}\;{\overset{\to \tau \;}{H^{\phi }}}^{T}+{\bar{W}}^{\tau }diag\left(\sum_{\tau }1\left(\left(\left(\overset{↑\phi \;}{V}\cdot \overset{↑\phi \;}{{\bar{\Lambda }}^{\left(\beta -2\right)}}\right)\;{\overset{\to \tau \;}{H^{\phi }}}^{T}\right)\cdot {\bar{W}}^{\tau }\right)\right)}\right]}^{-\delta (\beta )}$$

for $\tau =0,\;1,\;\ldots ,\;\tau_{max}$. By using the same approach, we can obtain the update for the sparse H as follows:

(61) $$H^{\phi }=H^{\phi }\cdot {\left[\frac{\sum_{\tau }{\overset{↓\phi \;}{{\bar{W}}^{\tau }}}^{T}\left(\overset{\leftarrow \tau \;}{V}\cdot \overset{\leftarrow \tau \;}{{\bar{\Lambda }}^{\left(\beta -2\right)}}\right)}{\sum_{\tau }{\overset{↓\phi \;}{{\bar{W}}^{\tau }}}^{T}\overset{\leftarrow \tau \;}{{\bar{\Lambda }}^{\left(\beta -1\right)}}+\alpha \lambda^{\phi }}\right]}^{\delta \left(\beta \right)}$$

for $\phi =0,\;1,\;\ldots ,\;\phi_{max}$. In Equation (61), α assumes the role of a regularization constant to balance the cost function fit and smoothness of H. In this work, we set α∈[0.5, 1] which has been found to give satisfactory results.

### 2.5. Optimizing the Fractional β

To determine the optimal value for *β*, we perform the investigation from the source separation viewpoint. Mathematically, the single-channel signal separation (SCSS) [37,38,39] problem can be treated as one mixture of N unknown source signals:

(62) $$y\left(t\right)=x_{1}\left(t\right)+x_{2}\left(t\right)+\ldots +x_{N}\left(t\right)$$

where t=1, 2, …, T denotes time index and the goal is to estimate the sources $x_{k}\left(t\right),\;\forall k\in N$ of length T when only the observation signal y(t) is available. For simplicity, we consider only N=2 sources in the mixture. We also use 50 different pieces of piano music, 50 different pieces of trumpet music and 50 different pieces of violin music from the RWC [40] database to generate different mixtures. The signal-to-distortion (SDR) [41] is used to measure the performance. The SDR results and its corresponding *β* value that produces the best performance in the separation of mixtures are shown in Table 2. From these experiments, we can propose some general ideas of how to choose a suitable *β* value: (i) The mixtures from same type of music share similar *β* value, e.g., the best results of piano and trumpet mixture occur around *β* = 2 but the best results of piano and violin mixture occur around β=1. (ii) If the power of one source is clearly weaker than the other source, then a smaller *β* value should be selected. (iii) When there is a large amount of overlap between the two sources in the in the time–frequency domain, a larger *β* value should be selected.

Table 2 strongly suggests that the *β* value depends on the mixture of original sources. Generally, it depends mainly on the two factors: (i) the weight of each source in the mixture; and (ii) the frequency spread of each source which the frequency band contains most weight of the signal. Firstly, we define weight of each source in the mixture by the following function:

(63) $$\gamma_{k}=1-\frac{\left|x_{k}\left(t\right)-y\left(t\right)\right|}{\sum_{l=1}^{N}{\left|x_{l}\left(t\right)-y\left(t\right)\right|}^{2}}$$

for k=1,…,N. The term $\gamma_{k}$ is nonnegative and bounded to unity. It measures the dominance of k-th source in the mixture. The higher value of $\gamma_{k}$, the greater the contribution from the k-th source is to the mixture. Secondly, we consider the separability of each source in the time–frequency domain by the following function:

(64) $$\eta_{k}=\frac{||M_{k}\left(i,j\right)X_{k}{\left(i,j\right)||}_{F}^{2}-||M_{k}\left(i,j\right)\sum_{l=1,l\neq k}^{N}X_{l}{\left(i,j\right)||}_{F}^{2}}{||X_{k}{\left(i,j\right)||}_{F}^{2}}$$

where $||\cdot {\left|\right|}_{F}$ is the Frobenius norm, $X_{k}\left(i,j\right)$ is the short-time Fourier Transform (STFT) of $x_{k}\left(t\right)$ with i representing the frequency bins and j the timeslot, and $M_{k}\left(i,j\right)$ is the binary mask Obtained from the k-th source as

(65) $$M_{k}\left(i,j\right)=\{\begin{array}{cc} 1 & \mathrm{if}{\left|X_{k}\left(i,j\right)\right|}^{2}>{\left|X_{l}\left(i,j\right)\right|}^{2}\\ 0 & \mathrm{otherwise} \end{array}$$

The function $\eta_{k}$ is also nonnegative and determines the degree separability of the signal in each frequency band. Based on the experiments conducted, both $\gamma_{k}$ and $\eta_{k}$ have an inverse relation to β. Thus, one possible empirical approach to determine β is proposed as follows:

(66) $$\beta \left(n+1\right)=\rho \left(n\right)\beta \left(n\right)+\left(1-\rho \left(n\right)\right)\min\left[\left(\sum_{k=1}^{N}\frac{\varepsilon_{1}\cdot \eta_{k}+\left(1-\varepsilon_{1}\right)\cdot \gamma_{k}}{\gamma_{k}\eta_{k}}\right),\;\varepsilon_{2}\right]$$

where ρ(n) is step size, $\varepsilon_{1}$ is a constant to weight the effects of $\eta_{k}$ and $\gamma_{k}$. For example, in the experiments conducted, we have given more emphasis to $\gamma_{k}$ and set $\varepsilon_{1}=1/3$. The term $\varepsilon_{2}$ is a constant to control the value of β(n+1) to ensure its value is bounded within an interval chosen by the user (for example, in the experiments conducted we have set $\varepsilon_{2}=4$ as normally does not exceed 4). Equation (66) is inserted into the update funtions in Equations (60) and (61) to update β at every iteration in conjunction with the update of W and H. In this case, β can be optimized based on the type of sources and the separation process. This enables the separation process to be fully automated and enables more accurate performance. In the case of SCSS, the sources are unknown and these are estimated from the mixture as:

(67) $${\hat{X}}_{k}\left(i,j\right)=M_{k}\left(i,j\right)Y\left(i,j\right)$$

where

(68) $$M_{k}\left(i,j\right)=\{\begin{array}{c} 1\;if\;{\left|{\overset{ˇ}{X}}_{k}\left(i,j\right)\right|}^{2}>{\left|{\overset{ˇ}{X}}_{l}\left(i,j\right)\right|}^{2}\\ 0\;otherwise\; \end{array}$$

and

(69) $${\left|{\overset{ˇ}{X}}_{k}\left(i,j\right)\right|}^{2}=\overset{\tau_{max}}{\underset{\tau =0}{\sum }}\overset{\phi_{max}}{\underset{\phi =0}{\sum }}{\bar{W}}_{i-\phi ,k}^{\tau }H_{k,j-\tau }^{\phi }$$

The expression in (69) is computed using the time–frequency deconvolution model. The main steps of the proposed algorithm have been summarized in Algorithm 1.

**Algorithm 1.** Overview Proposed Algorithm
**1.** Initialize $W^{\tau }$ and $H^{\phi }$ with non-negative random values.**2.** Compute the STFT:   Y(i,j)=STFT(y(t)), and let $V_{i,j}={\left|Y\left(i,j\right)\right|}^{2}$.**3.** Compute ${\bar{\Lambda }}_{i,j}=\sum_{k,\tau ,\phi }{\bar{W}}_{i-\phi ,k}^{\tau }\;H_{k,j-\tau }^{\phi }$.**4.** Compute $u_{p}\leftarrow u_{p}\left\{\left(-b_{p}+\sqrt{b_{p}^{2}+4\frac{{\left(Cu\right)}_{p}}{u_{p}}}\right)/2{\left(Cu\right)}_{p}\right\}$**5.** Assign $\lambda_{k,j}^{\phi }=\frac{1}{H_{k,j}^{\phi }+\delta_{k,j}^{\phi }}$ where $\delta_{k,j}^{\phi }=\{\begin{array}{c} \;0\;\mathrm{if}\;H_{k,j}^{\phi }\neq 0\;\\ u_{k,j}^{\phi }\;\mathrm{if}\;H_{k,j}^{\phi }=0 \end{array}$**6.** Update $H^{\phi }=H^{\phi }\cdot {\left[\frac{\sum_{\tau }{\overset{↓\phi \;}{{\bar{W}}^{\tau }}}^{T}\left(\overset{\leftarrow \tau \;}{V}\cdot \overset{\leftarrow \tau \;}{{\bar{\Lambda }}^{\left(\beta -2\right)}}\right)}{\sum_{\tau }{\overset{↓\phi \;}{{\bar{W}}^{\tau }}}^{T}\overset{\leftarrow \tau \;}{{\bar{\Lambda }}^{\left(\beta -1\right)}}+\alpha \lambda^{\phi }}\right]}^{\delta \left(\beta \right)}$**7.** Compute ${\bar{\Lambda }}_{i,j}=\sum_{k,\tau ,\phi }{\bar{W}}_{i-\phi ,k}^{\tau }\;H_{k,j-\tau }^{\phi }$.Update the spectral bases:**8.** $$W^{\tau }={\bar{W}}^{\tau }\cdot [\frac{\sum_{\phi }\left(\overset{↑\phi }{V}\cdot \overset{↑\phi }{{\bar{\Lambda }}^{\left(\beta -2\right)}}\right){\overset{\to \tau }{H^{\phi }}}^{T}+{\bar{W}}^{\tau }diag\left(\sum_{\tau }1\left(\left(\overset{↑\phi }{{\bar{\Lambda }}^{\left(\beta -1\right)}}{\overset{\to \tau }{H^{\phi }}}^{T}\right)\cdot {\bar{W}}^{\tau }\right)\right)}{\sum_{\phi }\overset{↑\phi }{{\bar{\Lambda }}^{\left(\beta -1\right)}}{\overset{\to \tau }{H^{\phi }}}^{T}+{\bar{W}}^{\tau }diag\left(\sum_{\tau }1\left(\left(\left(\overset{↑\phi }{V}\cdot \overset{↑\phi }{{\bar{\Lambda }}^{\left(\beta -2\right)}}\right){\overset{\to \tau }{H^{\phi }}}^{T}\right)\cdot {\bar{W}}^{\tau }\right)\right)}]$$**9.** For k=1,…,N, compute: $$\begin{array}{l} {\left|{\overset{ˇ}{X}}_{k}\left(i,j\right)\right|}^{2}=\overset{\tau_{max}}{\underset{\tau =0}{\sum }}\overset{\phi_{max}}{\underset{\phi =0}{\sum }}{\bar{W}}_{i-\phi ,k}^{\tau }H_{k,j-\tau }^{\phi }\\ M_{k}\left(i,j\right)=\{\begin{array}{c} 1\;if\;{\left|{\overset{ˇ}{X}}_{k}\left(i,j\right)\right|}^{2}>{\left|{\overset{ˇ}{X}}_{l}\left(i,j\right)\right|}^{2}\\ 0\;otherwise\; \end{array}\\ {\hat{X}}_{k}\left(i,j\right)=M_{k}\left(i,j\right)Y\left(i,j\right)\\ {\hat{x}}_{k}\left(t\right)={\mathrm{STFT}}^{-1}\left[{\hat{X}}_{k}\left(i,j\right)\right]\\ \gamma_{k}=1-\frac{\left|{\hat{x}}_{k}\left(t\right)-y\left(t\right)\right|}{\sum_{l=1}^{N}{\left|{\hat{x}}_{l}\left(t\right)-y\left(t\right)\right|}^{2}}\\ \eta_{k}=\frac{M_{k}\left(f,t\right){\hat{X}}_{k}{\left(f,t\right)}_{F}^{2}-M_{k}\left(f,t\right)\sum_{l=1,l\neq k}^{N}{\hat{X}}_{l}{\left(f,t\right)}_{F}^{2}}{{\hat{X}}_{k}{\left(f,t\right)}_{F}^{2}}\\ \beta \leftarrow \rho \beta +\left(1-\rho \right)\min\left[\left(\overset{N}{\underset{k=1}{\sum }}\frac{\varepsilon_{1}�\eta_{k}+\left(1-\varepsilon_{1}\right)�\gamma_{k}}{\gamma_{k}\eta_{k}}\right),\;\varepsilon_{2}\right] \end{array}$$**10.** Repeat Steps 3–9 until it converges or reaches the pre-defined number of iteration.


## 3. Experiments, Results and Analysis

In this section, we conduct in-depth investigations of the proposed algorithm to analyze the impact of fixed and adaptive sparsity, the adaptive behavior of the sparsity parameter, and the analysis of fractional *β*-divergence. The analysis is necessary as the issue of sparsity of the temporal codes would undermine the quality of matrix factorization when the β value is inappropriately chosen. The selection of the β value should consider the sparseness constraint used in the cost function. In addition, the proposed algorithm based on matrix factor time–frequency deconvolution is also compared to conventional NMF models. This allows us to quantify the impacts of fractional *β*-divergence and sparsity behaviors when using the time–frequency deconvolution model.

### 3.1. Experimental Set-Up

To investigate the proposed method, we use the algorithm to separate several pieces of mixed music signals. Several experimental simulations under different conditions have been designed to investigate the efficacy of the proposed method. All simulations were performed using MATLAB as the programming platform and performed using a PC with dual core processor @ 2.4 GHz (i7 Intel processor) 8 GB RAM and 320 GB HDD. The tested signals are generated by mixing several music sources. The polyphonic music is 4 s long and the sampling frequency is 16 kHz. In this experiment, we randomly chose 50 different pieces of piano music, 50 different pieces of trumpet music and 50 different pieces of violin music from the RWC database to produce the different mixtures. The mixed signal was then generated by adding the chosen sources. In all cases, the sources were mixed with equal average power over the duration of the signals. The time–frequency (TF) representation was obtained by first normalizing the time-domain signal to unit power and then by computing the STFT using 2048 point Hanning window FFT with a 50% overlap. We evaluated our separation performance in terms of the signal-to-distortion ratio (SDR) which is one form of perceptual measure. This is a global measure that unifies source-to-interference ratio (SIR), source-to-artifacts ratio (SAR) and source-to-noise ratio (SNR). The definition and mathematical expression and MATLAB routines for computing these criteria can be obtained online [42].

### 3.2. Analysis of Adaptive and Fixed Sparsity

**Figure 1:**
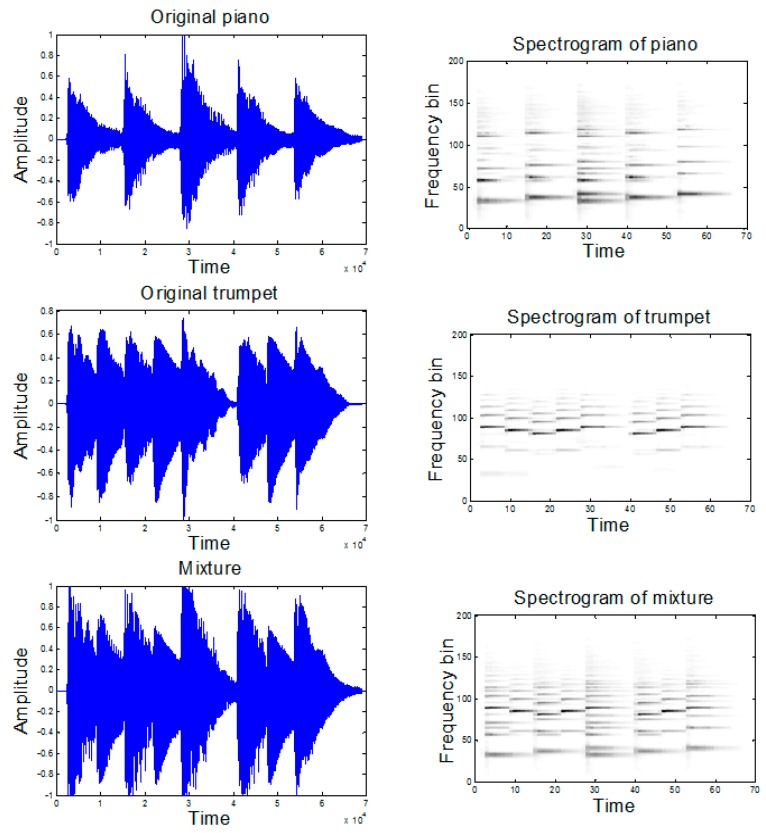
Time-domain representation and log-frequency spectrogram of: the piano music (**top panels**); the trumpet music (**middle panels**); and the mixed signal (**bottom panels**).

In this implementation, we conducted several experiments to compare the performance of the proposed method using different β values. Our aim was to investigate the impact of β value used in the separation. Figure 1 shows the time and TF domains of the original trumpet, piano music and its mixture. The TF domain is displayed using the log-frequency spectrogram. The trumpet and the piano play a different short melodic passage each consisting of three distinct notes. However, both trumpet and piano overlap in time, and the piano notes are interspersed in frequency with the trumpet notes. Hence, this is a challenging task for single channel separation which tests the impact of flexible β for matrix factorization.

**Figure 2:**
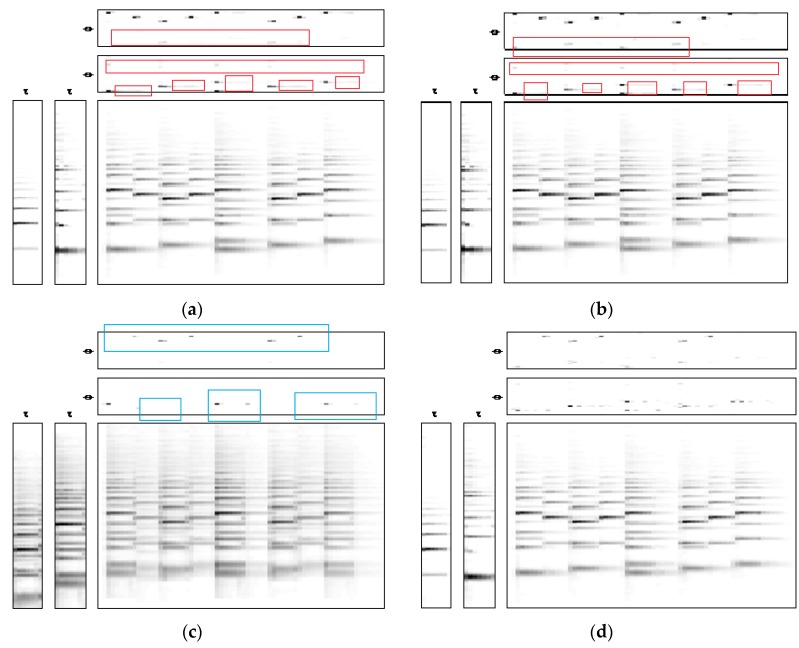
Estimation of $W^{\tau }$ and $H^{\phi }$ with: (**a**) λ=0; (**b**) λ=0.1; (**c**) λ=100; (**d**) λ=adaptive.

Figure 2 shows the estimation of bases $W^{\tau }$ and temporal codes $H^{\phi }$ when using different λ values. In Figure 2a, we set λ=0 which renders non-sparse solution. There is obvious spreading of the estimated temporal codes, as shown in the red part of the figure. In Figure 2b, when λ=0.1, there are some improvements over the spreading but they still exist in the red parts. Here, the sparseness is not strong enough and, as a result, the estimated mixture becomes under-sparse. In Figure 2c, when λ=100, it is visibly shown in the blue parts that some information has been lost in the estimated temporal codes and the resulting estimated mixture becomes very noisy. Finally, Figure 2d shows the case where the sparseness parameters are adaptively and individually estimated using the prior information of H. The obtained result has shown that the estimated temporal codes are just appropriately sparse and, by visual inspection, the resulting estimated mixture retains all information, as evidenced by the musical notes, while the noise level has been kept small, which very closely resembles the original mixture.

### 3.3. Adaptive Behavior of Sparsity Parameter

**Figure 3:**
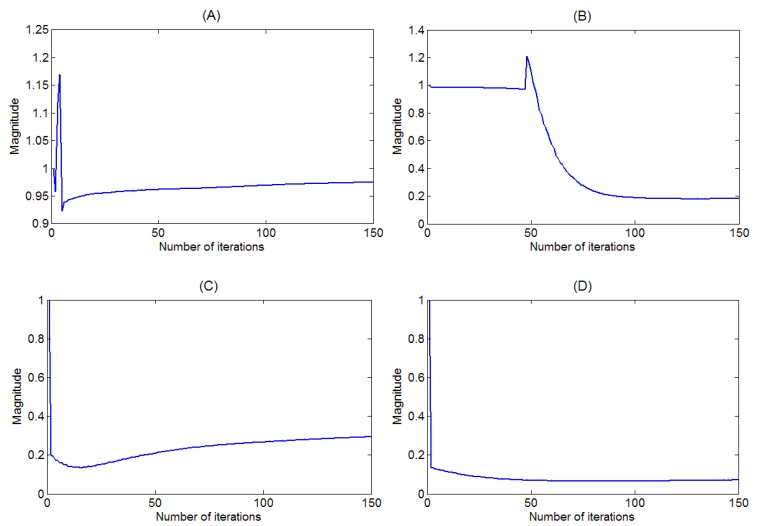
Convergence trajectory of the sparsity parameter: (**A**) $\lambda_{1,1}^{\phi =0}{}^{\;}$; (**B**) $\lambda_{1,5}^{\phi =0}$; (**C**) $\lambda_{1,10}^{\phi =0}$; and (**D**) $\lambda_{1,15}^{\phi =0}$.

In this section, we use the piano and trumpet mixture, and fix β value to 1 and the λ value ranges from 0 to 3 with each step of 0.1 to show the results. The adaptive behavior of the sparsity parameters using the proposed method is demonstrated. Figure 3 presents the convergence trajectory of four adaptive sparsity parameters, $\lambda_{1,1}^{\phi =0}$, $\lambda_{1,5}^{\phi =0}$, $\lambda_{1,10}^{\phi =0}$ and $\lambda_{1,15}^{\phi =0}$, corresponding to their respective element codes, $h_{1,1}^{\phi =0}$, $h_{1,5}^{\phi =0}$, $h_{1,10}^{\phi =0}$ and $h_{1,15}^{\phi =0}$. All sparsity parameters are initialized as λ=1. After 150 iterations, the above sparsity parameters converge to their steady-states. By examining Figure 3, it is noted that the converged steady-state values are significantly different for each sparsity parameter, e.g., $\lambda_{1,1}^{\phi =0}=0.9$, $\lambda_{1,5}^{\phi =0}=0.18$, $\lambda_{1,10}^{\phi =0}=0.29$ and $\lambda_{1,15}^{\phi =0}=0.08$, even though they started at the same initial condition. This shows that each element code has its own sparseness.

**Figure 4:**
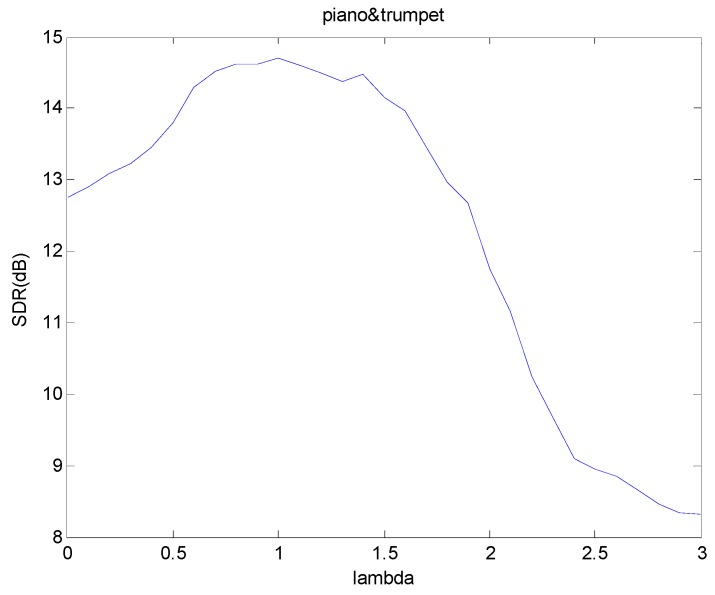
SDR results of piano and trumpet mixture when using different λ values.

In Figure 4, we compare the SDR results of using different λ values. In the figure, we can see the λ value that can get the best result changes with the mixture. For each different mixture, the best λ values are different. In Figure 4, we can see the best separation results of piano and trumpet mixture occurs near λ=1, and the SDR = 14.7 dB. However, as λ increases, the SDR performance begins to deteriorate rapidly due to over-sparseness of the temporal code $H^{\phi }$.

**Figure 5:**
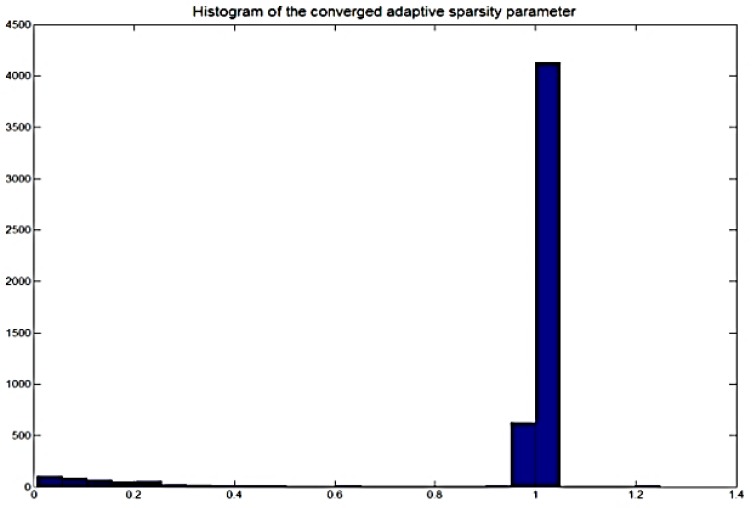
Histogram of the converged adaptive sparsity parameter.

Although the SDR performance in Figure 4 seems to suggest good performance, it may not necessary refer to the optimum setting. In fact, when λ is fixed to a constant value, the matrix factors deconvolution process may still be subjected to under- and over-fitting. In the previous sparsity algorithm, λ is fixed for the whole process and this sparsity may not necessarily suited for the whole signal. This calls for the need to allow each temporal code to have its own sparsity parameter. In the adaptive sparsity algorithm in Equation (53), the sparsity parameter λ is updated alongside $W^{\tau }$ and $H^{\phi }$ in the process. Therefore, the sparsity parameter is optimized for each element of the temporal code. In addition, we plot the histogram of the converged adaptive sparsity parameters in Figure 5. The plot strongly suggests that the histogram can be represented as a bimodal distribution. We have used the Gaussian mixture model (GMM) [43] to learn the distribution of this histogram and the result produces two Gaussian distributions with mean 0.16 and 1.1. The global mean of the GMM is given by 0.92.

With the GMM analysis, we can proceed to further investigate the assignment of sparsity parameters and compare them with the adaptive approach. We considered the following sparseness assignments:

Case (1): No sparseness λ=0.
Case (2): Uniform and constant sparseness λ=0.16 corresponding to the mean of the first Gaussian distribution of the GMM.
Case (3): Uniform and constant sparseness λ=1.1 corresponding to the mean of the second Gaussian distribution of the GMM.
Case (4): Uniform and constant sparseness λ=0.92 corresponding to the global mean of the converged adaptive sparsity.
Case (5): Uniform and constant sparseness λ=1 corresponding to the global mean of the converged adaptive sparsity.
Case (6): Maximum likelihood adaptive sparseness, i.e., Equation (55).

The SDR results are tabulated in Table 3 where we can see the separation results of all the six cases. The obtained results readily informed that the source separation with adaptive sparsity has rendered the best separation result.

### 3.4. Analysis of Fractional β-Divergence

**Figure 6:**
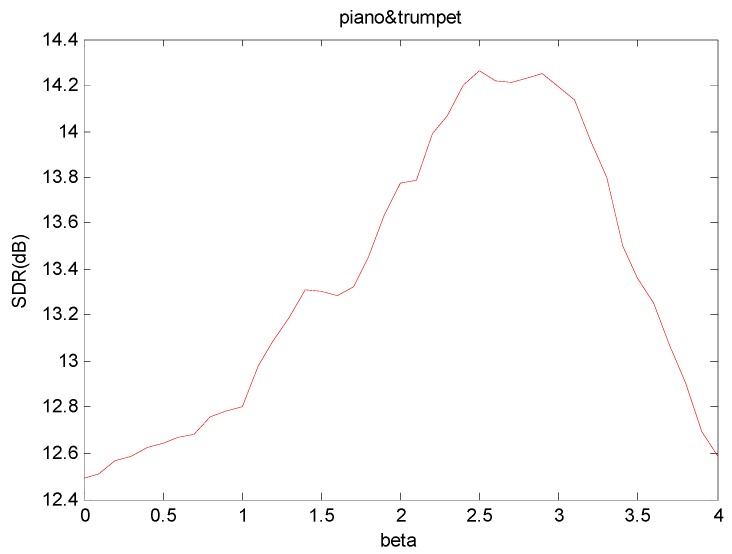
SDR values of the separation results of mixture using different *β* values.

Figure 6 shows the SDR values of the separation results using different values of *β* values. In this implementation, we fixed the sparsity parameter λ to 0, and the value of *β* ranges from 0 to 4, and each step is 0.1. In the figure, we can observe that the SDR value changes as the β value is increased. The best result is obtained at when β=2.5, where SDR = 14.26 dB. The SDR value keeps increasing for values of β value within the range of 0≤β<2.5, and, after the best performance is attained, the performance deteriorates as *β* increases. In this figure, we can see that the best performance does not necessarily occur at value of β used other algorithms, i.e., β=2, 1, 0. This means, if we choose the best *β* to carry out the separation, we can obtain better results than the other algorithms.

**Figure 7:**
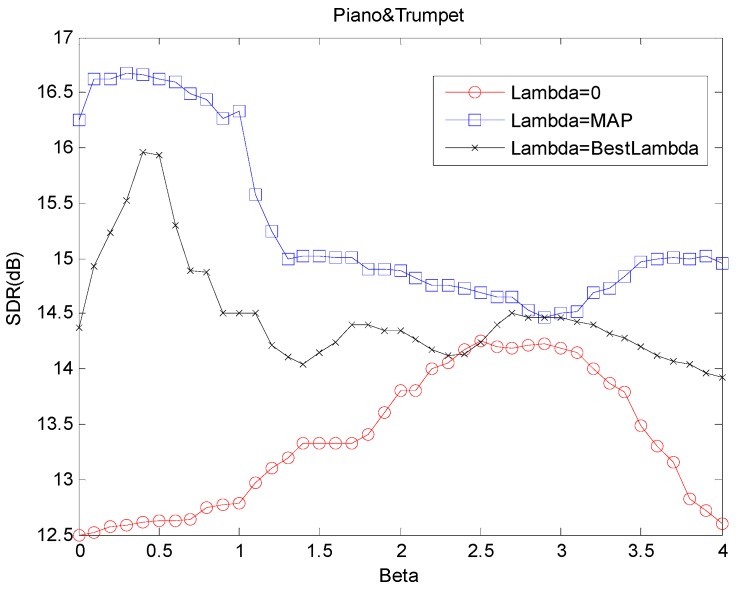
SDR values of the separation results of mixture using different *β* values and sparse methods.

We also conducted several experiments to compare the performance of the proposed method with the non-sparse and normal sparse methods using different β value. To investigate the impact of β and λ value used in the separation, we used β that ranges from 0 to 4 (with every increment of 0.1), and λ was set to several configurations, i.e., non-sparse, best fixed sparsity that is obtained in Figure 4 and the proposed adaptive sparsity. The results are plotted in Figure 7, which shows the mutual influence between *λ* and λ. It is noted that the SDR performance of non-sparse algorithm is the lowest of which its best result occurs when β=2.5 with SDR = 14.26 dB. The normal sparse algorithm using best λ value gives better performance than the non-sparse method with its best result occuring when β=0.5 with SDR = 15.96 dB. On the other hand, the proposed algorithm with adaptive λ delivers the best performance where β occurs around 0.3 with SDR = 16.71 dB. It is also noted that the worst SDR performance given by β=2.9 with adaptive λ is still higher than the highest SDR when β=2.5 without sparsity optimization λ=0.

All the above experiments used the pre-determined *β* that are fixed for the whole process. In this section, we present the results of experiments where β is adaptively tuned alongside with the adaptation of $W^{\tau }$, **H**^ϕ^ and $\lambda^{\phi }$. We applied this method to the source separation problem and compared the results with the situation where β=1 and β=2 corresponding to the Kullback–Leibler divergence and Least Square cost function, respectively. In the update of adaptive β, the step size $\rho \left(n\right)=0.95^{n}$ is selected which represents an exponential decay update process. The SDR results of the top five best performance are tabulated in Table 4. It is interesting to note from the table that the proposed algorithm with adaptive β delivers better performance by about 2.1 dB compared to that when β=1 (Kullback–Leibler divergence) and 1.9 dB compared to that when β=2 (Least Square distance). The obtained performance improvement is attributed to the fact that the joint optimization of *β* with $W^{\tau }$, $H^{\phi }$ and $\lambda^{\phi }$ has enabled the current estimate of β to better fit mixture of sources and thus rendered better source separation performance.

### 3.5. Comparison with Other Nonnegative Factorization Models

In this section, we compare the proposed algorithm with other signal separation algorithms, in both time-domain representation and analysis the SDR results of all algorithms. The signal chosen was the same piano and trumpet mixture music used in Section 3.4. We compared the Least Square NMF (NMF-LS) and Kullback–Leibler NMF (NMF-KLD) algorithms introduced in the earlier sections of this paper, NMF with temporal continuity and sparseness criteria (NMF-TCS) [23], and NMF with automatic relevance determination (NMF-ARD) [44]. The obtained results are summarized in Table 5.

When using the various NMF models, it is seen that the average improvement per source of about 3 dB has been gained by leveraging the fractional β value and adaptive sparsity. In addition, a step jump of approximately 5–8 dB in performance improvement is further obtained when the model is switched to the matrix factor time–frequency deconvolution. This is attributed to the latter model which represents both temporal structure and the pitch change which occur when an instrument plays different notes. The pitch change corresponds to a displacement on the frequency axis. Where NMF methods needed one component to model each note for each instrument, the time–frequency deconvolution model represents each instrument compactly by a single time–frequency profile convolved in both time and frequency by a time–pitch weight matrix [45]. The model dramatically decreases the number of components needed to model various instruments and effectively solves the blind single channel source separation problem for certain classes of musical signals.

## 4. Conclusions

This paper presents an adaptive fractional *β*-divergence with sparsity-aware optimization for non-negative factor time–frequency deconvolution algorithm. The impetus behind this work is that the previous β-divergence algorithms are all limited to special cases of β, and the previous sparsity methods are limited to a fixed sparsity parameter which are determined manually. Thus, these algorithms may not always produce the best results. In the proposed method, β is made adaptive and takes on fractional value. The sparsity parameter is also concurrently updated along with the estimation of β and model parameters. The convergence is theoretically proven for any β based on the auxiliary function method. This paper has shown that the proposed method is more general and can deliver better performance than other algorithms, as demonstrated using real audio recordings.

## Figures and Tables

**Table 1:** Differentiable Convex-Concave-Constant Decomposition of β-Divergence.

Range	$\overset{ˇ}{d}\left(y|x\right)$	$\hat{d}\left(y|x\right)$	$\bar{d}\left(x\right)$
β<1 and β≠0	$-\frac{1}{\beta -1}xy^{\beta -1}$	$\frac{1}{\beta }y^{\beta }$	$y^{\beta -1}$
β=0	$xy^{\beta -1}$	logy	$y^{-1}$
1≤β≤2	$d_{\beta }\left(x|y\right)$	0	0
β>2	$\frac{1}{\beta }y^{\beta }$	$-\frac{1}{\beta -1}xy^{\beta -1}$	$-xy^{\beta -2}$

**Table 2:** Results using different sources.

Mixtures	SDR (dB)	*β*
Piano + trumpet	16.11	2.11
	9.19	2.13
	9.43	1.93
	7.73	1.82
	12.21	2.09
Piano + violin	13.07	1.07
	8.15	1.23
	6.25	0.92
	9.33	1.20
	8.19	0.89
Trumpet + violin	14.63	0.68
	8.14	0.62
	7.81	0.67
	9.81	0.51
	7.55	0.52

**Table 3:** Results of separation for different mixture.

Methods	SDR (dB)
Case (1)	12.77
Case (2)	13.01
Case (3)	14.60
Case (4)	14.62
Case (5)	14.70
Case (6)	15.60

**Table 4:** SDR (dB) results of adaptive β versus fixed β.

Mixtures	SDR (dB) Using Adaptive *β*	SDR (dB) Using *β* = 1	SDR (dB) Using *β* = 2
Piano + Trumpet	16.85	14.11	15.93
	10.74	7.95	9.01
	9.93	8.12	9.11
	8.95	6.57	7.44
	13.64	10.26	12.03
Piano + Violin	14.17	12.12	11.67
	9.04	7.95	7.11
	8.13	6.09	5.81
	10.4	9.08	8.71
	9.59	7.85	7.19
Trumpet + Violin	15.40	12.49	12.13
	8.87	6.23	6.31
	9.14	6.87	7.17
	10.51	7.92	8.11
	9.17	7.77	7.95

**Table 5:** Performance comparison of proposed method with NMF models.

Algorithm	SDR (dB)
NMF-LS	4.17
NMF-KLD	3.47
NMF-TCS	5.12
NMF-ARD	3.98
NMF using proposed method	7.63
Proposed method using matrix factor time–frequency deconvolution	12.02
